# Building large-scale English–Romanian literary translation resources with open models

**DOI:** 10.3389/frai.2026.1807431

**Published:** 2026-06-17

**Authors:** Mihai Nadaş, Laura Dioşan, Andreea Tomescu, Andrei Pişcoran

**Affiliations:** 1 Department of Computer Science, Faculty of Mathematics and Computer Science, Babeş-Bolyai University, Cluj-Napoca, Romania; 2 KlusAI Labs, Cluj-Napoca, Romania

**Keywords:** literary machine translation, LLM-as-a-judge, LoRA, low-resource translation, parameter-efficient fine-tuning, Romanian, synthetic parallel data

## Abstract

**Introduction:**

Literary translation has recently gained attention as a distinct and complex task in machine translation research, yet translation by small open models remains an open problem, particularly for low-resource languages such as Romanian.

**Methods:**

We introduce the TinyFabulist Translation Framework (TF2), a unified framework for dataset creation, fine-tuning, and evaluation in English → Romanian literary translation. Building on DS-TF1-EN-3M, the largest collection of synthetic English fables to date, our pipeline first generates 15k high-quality Romanian references from the TF1 pool using a high-performing large language model (LLM). We then apply a two-stage fine-tuning process to a 12B-parameter open-weight model: (i) instruction tuning to capture genre-specific narrative style, and (ii) adapter compression for efficient deployment. Evaluation combines a five-dimension LLM-based rubric (accuracy, fluency, coherence, style, cultural adaptation) as the primary comparative framework, alongside corpus-level Bilingual Evaluation Understudy (BLEU) reported as a secondary reference-based consistency metric.

**Results:**

Our fine-tuned model (TF2-12B) achieves strong fluency and adequacy, narrowing the gap to top-performing proprietary models under automated and human-anchored evaluation, while being open, accessible, and significantly more cost-effective. We publicly release the fine-tuned model and two large-scale synthetic parallel datasets (DS-TF2-EN-RO-3M and DS-TF2-EN-RO-15K), along with all scripts and evaluation prompts.

**Discussion:**

TF2 provides an end-to-end, reproducible pipeline for research on cost-efficient translation, cross-lingual narrative generation, and the broad adoption of open models for culturally significant literary content in low-resource settings.

## 1 Introduction

Translating literature into low-resource languages poses unique challenges, given both the scarcity of parallel data and the need to faithfully preserve stylistic nuance and cultural context. Romanian—a Latin language spoken by over 24 million people—remains comparatively underserved in literary translation resources, especially relative to high-resource pairs such as English–French or English–German, with most publicly available EN–RO corpora concentrated in parliamentary, web, or news domains rather than creative narrative text. Existing machine translation (MT) benchmarks, such as Conference on Machine Translation (WMT),^1^ predominantly focus on informational or news text, thus providing limited guidance for creative, narrative-driven translation tasks. Recent advances in large language models (LLMs) have enabled synthetic text generation at unprecedented scale, opening new opportunities for augmenting MT datasets (Long et al., 2024; Nadas et al., 2025b). Notably, projects such as TinyStories (Eldan and Li, 2023) demonstrate that even compact models can generate coherent narratives when trained on carefully curated synthetic corpora. However, much of the existing work has focused on *monolingual* story generation in English (Guan et al., 2022; Eldan and Li, 2023) or on improvements to translation via back-translation (Sennrich et al., 2016), rather than on the systematic creation of large-scale bilingual literary datasets. As a result, open questions remain regarding how both proprietary and open-source LLMs can be leveraged to construct and evaluate literary translations in genuinely low-resource settings.

Substantial progress has also been made in building large bilingual and multilingual resources. For example, CLIRMatrix (Sun and Duh, 2020) compiles parallel data across 139 languages for cross-lingual retrieval, and mT5 (Xue et al., 2021) pretrains a text-to-text transformer on a wide range of language pairs and domains. These resources, however, primarily target general-domain or retrieval-oriented MT and include limited literary content. Even for language pairs such as English–Romanian, coverage is typically restricted to news, web, or miscellaneous domains. To date, no openly available, large-scale English–Romanian parallel corpus has been specifically curated for creative narratives or fables.

TINYFABULIST TRANSLATION FRAMEWORK (TF2) addresses this gap by extending our earlier TinyFabulist-TF1 corpus of English-only fables (Nadas et al., 2025a) into a bilingual setting. The resulting dataset, DS-TF2-EN-RO-3M , comprises three million synthetic English–Romanian parallel fables, accompanied by fine-tuned open-source translation models and a literary translation benchmark. By combining parameter-efficient model adaptation with a narrative-oriented evaluation protocol, TF2 provides both a large-scale resource and a practical experimental framework for studying literary machine translation in a low-resource language pair.

Recent multilingual models such as NLLB-200 (NLLB Team, 2022) and EuroLLM (Martins et al., 2025) achieve strong performance across many language pairs, but are generally optimized for breadth and general-domain translation. Their large parameter counts can also limit deployment in cost-constrained or on-device scenarios. In our experiments, these models do not consistently capture the stylistic nuance, discourse coherence, and cultural adaptation required for literary translation (Section 4), motivating the exploration of more targeted, domain-adaptive approaches.

Building on the English-only fable generation efforts of DS-TF1-EN-3M (Nadas et al., 2025a), we focus on moral fables—short narratives that require semantic fidelity alongside fluency, narrative coherence, stylistic faithfulness, and culturally appropriate moral framing. Generating parallel data for such content under resource constraints motivates the use of LLMs for both dataset construction and scalable evaluation.

Our contributions are summarized as follows:

- Narrative-Aware Evaluation Protocol. We introduce a multi-dimensional evaluation framework for literary translation that combines corpus-level Bilingual Evaluation Understudy (BLEU) (Sulem et al., 2018) with an LLM-based rubric assessing accuracy, fluency, coherence, style, and cultural adaptation. In this work, BLEU is used strictly as a secondary consistency check, while primary analysis relies on rubric-based and human-anchored evaluation.
- Two openly licensed corpora for literary MT. We release (i) DS-TF2-EN-RO-15K , a 15 000-example English → Romanian parallel corpus of moral fables, and (ii) DS-TF2-EN-RO-3M , a three-million-example corpus automatically translated using fine-tuned open models.^2^
- Three open, fine-tuned translation models. We release three LoRA-adapted English–Romanian translation models—tf2-1b, tf2-4b, and tf2-12b—which substantially narrow the performance gap to proprietary systems under cost-aware evaluation, while remaining efficient enough to run on commodity GPUs.^3^
- Fully transparent artifacts. All datasets, code, prompts, and evaluation scripts are released under permissive licenses to support reproducibility and further research in low-resource literary NLP.

### 1.1 Research questions

The construction of large-scale translation datasets and models is constrained by multiple practical considerations. Financial cost is often the most immediate limitation, particularly for low-resource language communities or research teams with restricted budgets. Additional factors include the environmental impact of large-scale computation—such as CO_2_ emissions and water use for hardware cooling—and the ethical provenance of training data, especially with respect to data privacy and fair representation. These considerations motivate the design of our experimental pipeline and inform the trade-offs examined in this work.

Accordingly, we structure our study around the following research questions:

RQ1: *Cost-Constrained Data Generation*: Can a large-scale English–Romanian literary translation dataset be constructed under strict budget constraints using small open models, and what trade-offs does this entail?
RQ2: *Open vs. Proprietary Translation Quality*: How do fine-tuned open models compare to proprietary systems for English–Romanian literary translation under cost-aware and resource-constrained settings?
RQ3: *Evaluation Rigor and Automation*: To what extent can an automated, LLM-based evaluation framework provide useful and interpretable signals for literary translation quality, when anchored by expert human judgment on a representative subset?

These research questions directly structure the contributions of the paper: RQ1 addresses cost-constrained dataset creation, RQ2 evaluates open vs. proprietary translation quality, and RQ3 examines the usefulness and limitations of automated, human-anchored evaluation.

The remainder of this paper is organized as follows. Section 2 reviews related work on literary machine translation, synthetic data generation, and evaluation. Section 3 describes the TF2 pipeline in detail. Section 4 presents experimental results and comparative evaluations. Section 5 introduces the released datasets and metadata schema. Sections 6 and 7 discuss findings and limitations, respectively.

## 2 Related work

### 2.1 Synthetic data for low-resource translation

Synthetic data has long been used to address data scarcity in machine translation, most notably through back-translation (Sennrich et al., 2016). Recent advances in large language models (LLMs) enable the scalable generation of monolingual and parallel corpora at substantially lower cost than manual annotation (Long et al., 2024; Wang et al., 2023). In narrative settings, synthetic corpora such as TINYSTORIES demonstrate that compact models can acquire coherent storytelling abilities when trained on carefully curated synthetic data (Eldan and Li, 2023). However, large-scale *bilingual* synthetic datasets targeting literary translation remain comparatively underexplored.

### 2.2 Narrative genres and moral fables

Short narrative forms, including moral fables, provide a controlled setting for studying discourse coherence, value alignment, and cultural framing. Datasets such as STORAL pair narratives with explicit morals and have been used for story understanding and moral reasoning tasks (Guan et al., 2022). Their structured yet expressive nature makes fables particularly suitable for scalable generation and evaluation in creative NLP, while remaining more tractable than longer literary forms.

### 2.3 Romanian NLP and translation resources

Romanian is included in several broader multilingual NLP and MT resources, including OPUS, Europarl, mT5, and NLLB-200, which support general-domain English–Romanian translation and cross-lingual transfer.Examples include OPUS (Tiedemann, 2012), Europarl (Koehn, 2005a), mT5 (Xue et al., 2021), and NLLB-200 (NLLB Team, 2022). However, these resources mainly focus on parliamentary, web, subtitle, or news text rather than literary narratives. Publicly available English–Romanian corpora specifically designed for creative translation remain limited, motivating the TF2 focus on fables and narrative style.

### 2.4 Open and proprietary translation models

Comparative studies show that proprietary LLMs typically outperform open-weight models for under-represented languages and creative domains, although this gap has narrowed with recent progress (Zheng et al., 2023). Parameter-efficient fine-tuning methods, such as LoRA (Hu et al., 2021), allow open models to be adapted to domain-specific tasks with modest computational cost. Large multilingual systems (e.g., NLLB-200 NLLB Team, 2022, EuroLLM Martins et al., 2025, DeepSeek-LLM DeepSeek-A et al., 2024) achieve strong general-domain translation performance, but their scale and optimization priorities limit accessibility and specialization for literary translation under cost constraints.

### 2.5 Evaluation beyond lexical overlap

BLEU (Papineni et al., 2002) remains a widely used MT metric, but its correlation with human judgment degrades for creative and stylistically rich text. Alternative frameworks such as MQM (Lommel et al., 2014) and COMET (Rei et al., 2020) provide more fine-grained quality signals, often at higher annotation or modeling cost. More recently, LLM-based evaluators have emerged as scalable proxies for human judgment, showing promising alignment across several text generation tasks (Liu et al., 2023). These approaches motivate the use of rubric-based, multi-dimensional evaluation for narrative translation.

## 3 TinyFabulist Translation Framework (TF2)

TF2 generalizes the original TINYFABULIST FRAMEWORK (TF) project to a bilingual and scalable setting, introducing an end-to-end methodology for constructing an English–Romanian literary translation resource. The framework emphasizes transparency, replicability, and cost-awareness, prioritizing open artifacts, parameter-efficient adaptation, and evaluation that combines automatic metrics, rubric-based assessment, and targeted human judgment.

**Figure 1:**
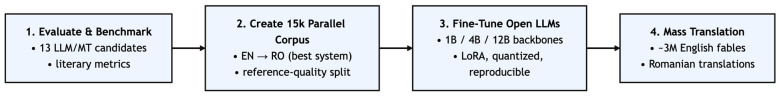
Overview of the TINYFABULIST TRANSLATION FRAMEWORK (TF2) pipeline. Stage 1 evaluates and benchmarks candidate translation systems on literary metrics. Stage 2 constructs a high-quality 15k English–Romanian parallel corpus using the top-ranked system. Stage 3 applies parameter-efficient fine-tuning of open LLMs (1B–12B backbones). Stage 4 performs large-scale translation of approximately 3M English fables into Romanian.

The workflow (see Figure 1) consists of four stages:

(a) Evaluating Candidate Translators: We benchmark 13 systems spanning proprietary LLMs (GPT, Gemini, Grok), commercial MT (DeepL), and open instruction-tuned models (EuroLLM, Gemma variants), listed in full in Table 1. Evaluation targets multiple aspects of literary translation and uses a five-dimension LLM-based rubric to compare systems in a scalable and consistent manner.
(b) Parallel Dataset Creation: The top-ranked Stage 1 system is used to translate 15,000 English fables, producing the TinyFabulist DS-TF2-EN-RO-15K corpus. This silver-standard parallel dataset provides supervision for downstream adaptation in a setting without human-authored references, and supplies a consistent reference corpus for subsequent reference-based evaluation.
(c) Parameter-Efficient Fine-Tuning: A suite of open LLMs—ranging from 1B to 12B parameters—are fine-tuned on the 15k parallel set using Low-Rank Adaptation (LoRA; Hu et al., 2021), yielding domain-adapted English–Romanian translation models. Quantized variants are produced to support efficient local inference.
(d) Large-Scale Corpus Generation: The best-performing fine-tuned model(s) are used to translate the remaining ~3M English fables from DS-TF1-EN-3M , resulting in a large bilingual English–Romanian corpus for Romanian literary MT. The pipeline is modular and supports future re-generation as translation models or evaluation protocols improve.

**Table 1:** Non-TF2 baselines on the TF2 literary translation benchmark.

Model	Accuracy	Fluency	Coherence	Style	Cultural	Avg. score	Count
**GPT-o3 (2025-04-16)**	**4.86**	**4.92**	**4.89**	**4.96**	**4.97**	**4.92**	100
GPT-4.1-mini (2025-04-14)	4.54	4.71	4.72	4.84	4.83	4.73	98
GPT-4.1 (2025-04-14)	**4.86**	4.89	4.85	4.92	4.94	4.89	100
GPT-o3-mini (2025-01-31)	4.71	4.78	4.87	4.85	4.92	4.83	100
Gemini-2.5-Flash	4.75	4.86	4.82	4.87	4.89	4.84	100
Gemini-2.0-Flash-001	4.66	4.82	4.78	4.89	4.93	4.82	100
Gemini-Flash-1.5-8b	4.14	4.45	4.67	4.52	4.46	4.45	99
DeepL	4.42	4.73	4.38	4.69	4.74	4.59	100
Grok-3-mini-beta	4.73	4.74	4.77	4.82	4.88	4.79	100
EuroLLM-9B-Instruct	3.84	4.27	4.36	4.27	4.22	4.19	98
Gemma-3-12B-it	3.98	4.56	4.65	4.52	4.43	4.43	100
Gemma-3-4B-it	3.27	3.94	4.17	3.91	3.78	3.81	100
Gemma-3-1B-it	1.79	2.13	2.23	2.07	1.86	2.02	100

Columns are rubric scores (max 5), their mean (Avg. Score), and sample count. Bold indicates the best value per rubric column. Each input sequence contains on average 300–400 tokens, with outputs averaging 350–450 tokens.

Each stage is integrated with quality control and open artifact release, providing a reproducible blueprint for low-resource literary MT beyond English–Romanian.

### 3.1 Stage 1: evaluating models and selecting translators

The first stage serves two objectives: (i) selecting a translation system to act as a reference translator for parallel corpus construction, and (ii) identifying a competitive open-source backbone suitable for downstream parameter-efficient fine-tuning. We evaluate a diverse pool of candidate systems, including commercial MT APIs, proprietary LLMs, and open instruction-tuned models, with a focus on English–Romanian literary translation.

Evaluation Methodology. Translation quality in literary narratives involves multiple complementary aspects, including semantic fidelity, linguistic well-formedness, discourse coherence, stylistic appropriateness, and cultural adaptation. To capture these facets in a scalable and comparable manner, we employ rubric-based LLM evaluation at Stage 1 and introduce reference-based metrics (e.g., BLEU) once a consistent reference corpus becomes available in later stages. Targeted human assessment is used to anchor the interpretation of automated scores (Section 4.5).

At Stage 1, where no human-authored Romanian references are available, candidate systems are compared primarily using a multi-dimensional rubric implemented via an LLM acting as an automated judge. This rubric evaluates five dimensions derived from professional translation guidelines:

1. Accuracy: Fidelity to the semantic content of the source text.
2. Fluency: Grammaticality, naturalness, and readability in the target language.
3. Coherence: Logical flow and clarity at the story level.
4. Style: Appropriateness of tone and consistency with the narrative voice.
5. Cultural/Pragmatic Adaptation: Appropriateness of cultural references and moral framing for the target audience.

Each dimension is scored on a 1–5 Likert scale (1 = poor, 5 = excellent). To operationalize this rubric at scale, we prompt an LLM to emulate a professional literary translation reviewer:

“*You are a professional translation evaluator. You will be given an English fable and a Romanian translation. Evaluate the translation for accuracy, fluency, coherence, style, and cultural/pragmatic fidelity. Provide a score from 1 to 5 for each category, along with a brief justification. Output your evaluation in valid JSON format with fields for each score and justification.”*

The structured JSON output enables reliable aggregation of scores across systems. For each candidate model, we evaluate a randomly sampled subset of 100 distinct fables, yielding per-dimension scores and short qualitative rationales.The 100-item evaluation subset was selected as a cost-efficient compromise between breadth and repeated API-based scoring expense. Pilot runs indicated that rankings stabilized at similar sample sizes, so 100 items were adopted uniformly across systems for comparability. The resulting averages provide a comparative view of translation behavior across systems. This approach follows prior work showing that LLM-based rubrics can provide useful comparative signals in text generation evaluation (Liu et al., 2023). In our pipeline, these scores are used as an auxiliary decision signal for model selection rather than as an absolute measure of translation quality.

A targeted pilot human substudy with one independent evaluator is later reported in Section 4.4 and is used only as qualitative anchoring evidence rather than formal statistical validation.

Selecting Translators and Backbones. Based on aggregated rubric scores, the highest-ranked system is selected as the reference translator for parallel corpus construction and downstream benchmarking. Independently, the strongest-performing open-source model is selected as the backbone for parameter-efficient fine-tuning in Stage 3.

### 3.2 Stage 2: parallel corpus generation (15k fables)

A central step in constructing a benchmark for low-resource literary MT is the creation of a parallel dataset tailored to the narrative and stylistic demands of the target genre. In TF2 , we address the shortage of English–Romanian literary data by leveraging LLMs to generate a diverse set of story translations, following established practices in synthetic corpus construction (Long et al., 2024; Eldan and Li, 2023).

We sample 15,000 short fables from DS-TF1-EN-3M ^4^, each consisting of a concise narrative ending with an explicit moral. We then translate them into Romanian using the top-ranked translator selected in Stage 1. This yields a machine-generated parallel corpus intended to serve as both a training resource and a consistent reference set for subsequent benchmarking.

The resulting TinyFabulist DS-TF2-EN-RO-15K dataset is released on Hugging Face^5^ under the permissive MIT License. Each record is stored in JSONL format and includes the English source, the Romanian translation, and metadata to support downstream analysis and reproducibility. Key fields include:

- fable: The original English fable text (typically 1–3 paragraphs), ending with an explicit moral.
- translated_fable: The Romanian translation generated by the selected model.
- pipeline_stage: Stage of the data-generation pipeline that produced the record (here translation).
- source_lang and target_lang: Language codes indicating source and target (here always English→Romanian).
- prompt_hash: SHA-256 hash of the translation prompt, used for reproducibility and traceability.
- llm_name: Identifier of the model used for translation (e.g., model name or Hugging Face snapshot).
- translation_model: Identifier of the specific checkpoint used to produce the translation (when distinct from llm_name).
- generation_timestamp: Unix timestamp indicating when the translation was generated.

The dataset is split into 12,000 training pairs, 1,500 validation pairs, and 1,500 test pairs. Although the Romanian references are model-generated, their selection follows the benchmarking procedure described in Section 3.1. The accompanying metadata supports reproducibility, comparative evaluation, and auditing of translation provenance.

A qualitative spot review of sampled entries indicated that the most common residual issues were minor lexical literalism, occasional stylistic flattening of morals, and rare punctuation inconsistencies, rather than severe semantic mistranslations.

Overall, TinyFabulist DS-TF2-EN-RO-15K provides a transparent foundation for training and evaluating English–Romanian literary MT models in the absence of large human-translated corpora.

### 3.3 Stage 3: fine-tuning—Parameter-efficient domain adaptation

The third stage adapts open instruction-tuned LLMs to the literary translation domain using parameter-efficient fine-tuning. Instead of updating all model parameters, we employ Low-Rank Adaptation (LoRA; Hu et al., 2021), which injects lightweight, trainable adapters into attention and feed-forward layers while keeping most parameters frozen. This approach enables domain adaptation with relatively modest computational overhead.

Training Methodology. The fine-tuning process follows established instruction-tuning practices:

1. Data preparation: Align and preprocess English–Romanian text pairs into instruction–response format, filtering for maximum length and domain relevance. Each input is prefixed with a standardized prompt (e.g., “Translate the following fable from English to Romanian:”).
2. Tokenization and masking: Employ the native SentencePiece or BPE vocabulary associated with each model family. In the labels tensor, mask all tokens except the Romanian output, ensuring that only target-side predictions contribute to the training loss.
3. Adapter injection: Attach LoRA adapters to major projection matrices in both self-attention and feed-forward blocks—q_proj, k_proj, v_proj, o_proj, gate_proj, up_proj, and down_proj. For each weight $W\in {\mathbb{R}}^{d_{\text{out}}\times d_{\text{in}}}$ we learn a low-rank update $\Delta W=\frac{\alpha }{r}BA$ with *r* = 32, α = 32 (effective scaling = 1.0), and apply dropout *p* = 0.05 on the adapter path. We train only *A, B* while freezing the backbone (no bias adaptation), and merge Δ*W* into *W* at inference. This follows best practices in recent literature (Mao et al., 2024; Ding et al., 2024).
4. Training regime: Fine-tune models using AdamW optimization, gradient accumulation, cosine learning rate scheduling, and mixed-precision (FP16 or bfloat16) arithmetic. To reduce overfitting, we apply early stopping based on held-out validation loss.
5. Deployment: After training convergence, merge LoRA adapters into the base weights for standalone inference and produce 8-bit quantized variants (Dettmers et al., 2022; Frantar et al., 2023) to reduce memory and latency. We additionally leverage llmcompressor to generate W8A8 quantized models, compatible with vLLM (Kwon et al., 2023; Shaw and Kurtz, 2024). For offline distribution we also export GGUF artifacts compatible with llama.cpp and Hugging Face GGUF endpoints^6^.

Evaluation. Fine-tuned models are assessed using both rubric-based evaluation (as in Stage 1) and BLEU. BLEU is reported as a secondary reference-based consistency metric by comparing translations against the reference corpus created in Stage 2, while primary comparative conclusions rely on the multidimensional rubric.

Accordingly, reference-based metrics are interpreted only as consistency relative to a strong silver-standard translator, not as absolute human-ground-truth adequacy.

Backbone Coverage. This fine-tuning procedure is applied across multiple model scales to assess robustness under varying computational constraints. Results are reported in Section 4, including changes in both rubric scores and BLEU relative to untuned backbones.

### 3.4 Stage 4: large-scale fable translation

The final stage translates the full TinyFabulist English corpus into Romanian using the fine-tuned models. DS-TF1-EN-3M comprises approximately 3 million AI-generated English fables, which we translate into Romanian primarily using the best-performing fine-tuned checkpoint. In addition, for a small number of runs we also generate translations with alternative fine-tuned checkpoints to support ablations and comparative analyses reported in Section 4. This process yields the DS-TF2-EN-RO-3M dataset, an openly released English–Romanian parallel corpus for literary translation research.

The translation process is designed to be feasible on modest hardware (e.g., small GPU clusters or standard cloud infrastructure) without prohibitive computational cost. The resulting dataset is released on the Hugging Face Hub.^7^

In addition, manual spot-checking was performed on randomly sampled outputs to verify formatting integrity, language consistency, and absence of obvious truncation or hallucinated content.

*A full statistical and structural overview of the* *DS-TF2-EN-RO-3M* *dataset—including metadata schema, field definitions, and example use cases—is provided in Section* *5*.

Complete hardware and software specifications are listed in Supplementary Appendix A.

## 4 Experiments and results

This section evaluates TF2 's translation models against a range of strong baselines—both proprietary and open—using reference-based metrics (BLEU), an LLM-based rubric, and a targeted human evaluation. We compare all fine-tuned TF2 models, their quantized variants, and the influence of decoding temperature on translation quality.

### 4.1 Benchmarked systems and baselines

We first benchmark only *external* systems as obtained “out of the box” from their public endpoints or hubs, with no additional fine-tuning. The pool spans:

- Proprietary LLMs and commercial MT: GPT-4.1, GPT-4.1-mini, GPT-o3, GPT-o3-mini, Gemini-2.5, Gemini-2.0, Grok-3, DeepL.
- Open instruction-tuned baselines: EuroLLM-9B, Qwen3-14B, and untuned Gemma-3 models (1B/4B/12B).

For each model we compute the per-dimension rubric and the overall average.


$$\begin{array}{c} \text{Avg. Score}=\frac{1}{5}\underset{d\in \left\{\text{Acc.},\text{Flu.},\text{Coh.},\text{Sty.},\text{Cult./Prag.}\right\}}{\sum }d. \end{array}$$ (1)

This aggregation is defined in Equation 1. Because GPT-o3 references and GPT-o3-mini judging originate from the same model family, stylistic preference bias cannot be fully excluded. We therefore conducted a cross-family re-evaluation using Grok-3-mini, which preserved system rankings.

#### 4.1.1 Why GPT-o3 as the reference translator?

Table 1 indicates that GPT-o3 attains the highest average rubric score across the evaluated systems. We therefore adopt GPT-o3 outputs as *silver-standard* references for reference-based comparisons elsewhere in the paper. Subsequent BLEU results use these GPT-o3 translations as references, which is why GPT-o3 itself does not appear with a comparable BLEU entry in those tables.

#### 4.1.2 Why Gemma-3-12B-it as the open backbone to fine-tune?

Among the open baselines, Gemma-3-12B-it achieves the highest rubric average (4.43), outperforming the 4B (3.81) and 1B (2.02) variants. It also offers a permissive license and mature tooling, making it a practical foundation for adaptation. We therefore select Gemma-3-12B-it (and, for scaling experiments, its 4B and 1B counterparts) as backbones for TF2 fine-tuning.

### 4.2 Overall translation quality with TF2 models

#### 4.2.1 TF2 fine-tuned models

The TF2 series comprises three parameter-efficient fine-tuned variants of the Gemma-3 instruction-tuned backbones: Gemma-3-1B-it, Gemma-3-4B-it, and Gemma-3-12B-it. Each model is adapted on the DS-TF2-EN-RO-15K corpus using LoRA adapters injected into all major projection matrices (q_proj, k_proj, v_proj, o_proj, gate_proj, up_proj, down_proj) with configuration *r* = 32, α = 32, and dropout *p* = 0.05. Training follows standard low-rank adaptation practice (Mao et al., 2024), with adapters merged into the base weights after convergence. For deployment, we provide 8-bit quantized variants, as well as a distilled TF2-1B checkpoint derived from teacher–student compression. This yields three self-hostable translation models—TF2-1B, TF2-4B, and TF2-12B—covering small, medium, and large parameter ranges.

We compare each fine-tuned TF2 model directly to its corresponding untuned backbone: Gemma-3-1B-it, Gemma-3-4B-it, and Gemma-3-12B-it. We also evaluate decoding robustness across temperatures *T*∈{0.0, 0.2, 1.0}.

We observe that BLEU scores and rubric-based evaluations do not always correlate monotonically across models and decoding settings. This divergence is expected in literary translation, where higher-quality outputs may involve paraphrasing, restructuring, and stylistic variation that reduce n-gram overlap with a single reference. Accordingly, we interpret BLEU primarily as a consistency signal relative to the chosen reference translation, and not as a proxy for overall literary quality.

Below, we summarize the key results for each parameter scale.

- TF2-1B (Gemma-3-1B-it backbone): After fine-tuning, the smallest model attains an average rubric score of 3.75 (up from 2.02). BLEU remains comparatively low (0.2180 for TF2-1B-distilled; 0.0543 for TF2-1B), consistent with the difficulty of long-form narrative translation for compact architectures.
- TF2-4B (Gemma-3-4B-it backbone): The 4B model achieves rubric averages above 4.5 across all temperatures, peaking at 4.74 (*T* = 0.0). BLEU ranges from 0.0496 (quantized, *T* = 0.2) to 0.1290 (quantized, *T* = 0.0) and 0.1278 (FP16, *T* = 1.0). Relative to the untuned backbone (avg. rubric 3.81, BLEU 0.1005), the fine-tuned variants show consistent gains under rubric-based evaluation.
- TF2-12B (Gemma-3-12B-it backbone): The largest model provides the strongest results among the open TF2 models. At *T* = 0.0, TF2-12B attains an average rubric score of 4.83 with BLEU 0.0926; at *T* = 0.2, 4.82 with BLEU 0.0647; and at *T* = 1.0, 4.66 with BLEU 0.0784. The 8-bit quantized version remains close in rubric scores across temperatures, consistent with prior findings on activation-aware quantization (Lin et al., 2024). These results improve substantially over the untuned Gemma-3-12B-it baseline (avg. rubric 4.43, BLEU 0.0214).

A summary of the main results—with both BLEU and LLM-based rubric scores—is provided in Tables 2, 3. For each model scale, we report the fine-tuned TF2 model, its quantized variant (where applicable), and the corresponding Gemma backbone for direct comparison.

**Table 2:** BLEU scores for each fine-tuned TF2 model and its corresponding Gemma backbone.

Model	BLEU score	Notes
TF2-12B (T = 0.0)	0.0926	Fine-tuned (FP16)
TF2-12B (T = 0.2)	0.0647	Fine-tuned (FP16)
TF2-12B (T = 1.0)	0.0784	Fine-tuned (FP16)
TF2-12B-quant (T = 0.0)	0.0746	Quantized (8-bit)
TF2-12B-quant (T = 0.2)	0.0644	Quantized (8-bit)
TF2-12B-quant (T = 1.0)	0.0548	Quantized (8-bit)
Gemma-3-12B-it	0.0214	Untuned base
TF2-4B (T = 0.0)	0.1153	Fine-tuned (FP16)
TF2-4B (T = 0.2)	0.1094	Fine-tuned (FP16)
TF2-4B (T = 1.0)	0.1278	Fine-tuned (FP16)
TF2-4B-quant (T = 0.0)	0.1290	Quantized (8-bit)
TF2-4B-quant (T = 0.2)	0.0496	Quantized (8-bit)
TF2-4B-quant (T = 1.0)	0.0857	Quantized (8-bit)
Gemma-3-4B-it	0.1005	Untuned base
TF2-1B	0.0543	Fine-tuned (T = 0.5)
TF2-1B-distilled	0.2180	Distilled
Gemma-3-1B-it	0.0790	Untuned base

Quantized and distilled variants are included. BLEU reflects test set n-gram overlap with the GPT-o3 reference and is reported on a normalized 0–1 scale (0.0926≡9.26 BLEU points on the conventional scale). Lower BLEU for Gemma-3-12B-it vs. Gemma-3-4B-it can arise from greater paraphrasing and structural divergence by the larger model, which BLEU penalizes despite higher rubric scores.

**Table 3:** Five-dimension LLM rubric evaluation for each fine-tuned TF2 model and corresponding Gemma backbone.

Model	Accuracy	Fluency	Coherence	Style	Cultural	Avg. score
TF2-12B (T = 0.0)	4.72	4.88	4.84	4.87	4.85	**4.83**
TF2-12B (T = 0.2)	4.69	4.88	4.83	4.88	4.83	4.82
TF2-12B (T = 1.0)	4.47	4.75	4.71	4.73	4.66	4.66
TF2-12B-quant (T = 0.0)	4.67	4.87	4.79	4.89	4.86	4.82
TF2-12B-quant (T = 0.2)	4.70	4.86	4.85	4.86	4.83	4.82
TF2-12B-quant (T = 1.0)	4.44	4.69	4.66	4.70	4.72	4.64
Gemma-3-12B-it	3.98	4.56	4.65	4.52	4.43	4.43
TF2-4B (T = 0.0)	4.64	4.76	4.71	4.80	4.79	4.74
TF2-4B (T = 0.2)	4.59	4.76	4.67	4.82	4.84	4.73
TF2-4B (T = 1.0)	4.39	4.59	4.56	4.74	4.66	4.59
TF2-4B-quant (T = 0.0)	4.60	4.76	4.74	4.81	4.79	4.74
TF2-4B-quant (T = 0.2)	4.49	4.75	4.64	4.76	4.79	4.69
TF2-4B-quant (T = 1.0)	4.21	4.53	4.56	4.60	4.58	4.50
Gemma-3-4B-it	3.27	3.94	4.17	3.91	3.78	3.81
TF2-1B-distilled	3.41	3.78	4.00	3.81	3.65	3.73
TF2-1B	3.43	3.80	4.02	3.83	3.67	3.75
Gemma-3-1B-it	1.79	2.13	2.23	2.07	1.86	2.02

Scores are averaged over a held-out test set. Bold values indicate the best (highest) score in each rubric column.

Each reported rubric average is based on approximately 500 individual dimension ratings per model (100 sampled fables × 5 dimensions). Because per-item score distributions are not reported here, small score gaps should be interpreted directionally rather than as formal significance tests. Importantly, the overall ranking patterns were stable across dimensions, the cross-family judge check in Table 4, and the pilot human evaluation.

**Table 4:** Cross-family LLM-as-judge robustness on the same 100-item subset and five-dimension rubric.

	GPT-o3-mini (judge)		Grok-3-mini (judge)		Avg. of judges
System	Score ↑	Gap to o3 ↓	Score ↑	Gap to o3 ↓	
o3 (reference translations)	4.92	0.00	4.92	0.00	4.92
TF2-12B-Q	4.82	0.10	4.90	0.02	4.86
TF2-12B	4.82	0.10	4.85	0.07	4.84

An unrelated judge (Grok-3-mini) reproduces the ranking and reduces the TF2–o3 gap relative to GPT-o3-mini.

#### 4.2.2 Cross-family judge bias check

Because our silver-standard references are produced by GPT-o3 and our primary evaluator is GPT-o3-mini (Table 1), we probe for possible *judge-family bias* by re-scoring the same 100-item subset with an unrelated judge, **Grok-3-mini**, using the same rubric, prompt, and randomized system order described in Section 3.1. As shown in Table 4, the system ranking is stable across judges. The TF2–o3 gaps are smaller under the alternative judge, suggesting that the main conclusions do not depend on a single judge family.^8^

##### 4.2.2.1 Takeaways

(1) The system ranking is stable across judges from different families. (2) The absolute TF2–o3 gaps are small ( ≤ 0.10 on a 1–5 rubric) and shrink further under Grok-3-mini (to 0.02–0.07). (3) The quantized TF2-12B-Q slightly exceeds TF2-12B under Grok-3-mini (4.90 vs. 4.85); we leave deeper ablations to future work.

### 4.3 Effect of decoding temperature

#### 4.3.1 Temperature sweep analysis

Ablation across decoding temperatures (*T*∈{0.0, 0.2, 0.5, 1.0}) reveals clear trends:

- Lower temperatures (*T* = 0.0, 0.2) yield the highest overall scores for both FP16 and quantized models, with optimal values observed at *T* = 0.0 for TF2-12B (4.83), TF2-4B (4.74), and their quantized versions (4.82, 4.74).
- Higher temperatures (*T* = 1.0) reduce performance across all models, with average rubric scores dropping by up to 0.2–0.3 compared to greedy decoding. This decline is most pronounced in Accuracy and Cultural/Pragmatic dimensions, consistent with increased hallucination and stylistic drift.
- Quantization robustness: 8-bit quantized checkpoints track their FP16 counterparts within 0.01–0.03 points, indicating limited loss under the rubric.

For subsequent large-scale translation runs, *T* = 0.0 is adopted as the default.

### 4.4 Human evaluation substudy

To complement the automated rubric-based evaluation, we conducted a targeted pilot human assessment designed to provide qualitative anchoring for the system rankings reported elsewhere in the paper.

One independent Romanian native speaker with academic training in linguistics and prior experience in literary text analysis evaluated a blinded subset of 40 randomly sampled fables drawn from the TF2 test set. The evaluator was not involved in model training, prompt engineering, metric design, or score aggregation.

For each English source fable, three Romanian candidate translations were presented in randomized and anonymized order, corresponding to: (i) GPT-o3 (reference system), (ii) TF2-12B (best open fine-tuned model), and (iii) Gemma-3-12B-it (untuned open baseline).

Translations were evaluated using the same five dimensions employed in the automated rubric:

- Accuracy: preservation of source meaning and factual content;
- Fluency: grammatical correctness, readability, and natural Romanian phrasing;
- Coherence: logical flow and narrative clarity across the story;
- Style: preservation of tone, voice, and literary appropriateness;
- Cultural Adaptation: natural rendering of morals, idioms, and pragmatic cues for Romanian readers.

Each dimension was scored on a 1–5 Likert scale. In addition, the evaluator selected an overall preferred translation for each source item and was encouraged to provide optional short qualitative comments when outputs differed substantially.

The resulting rankings broadly aligned with the automated rubric: GPT-o3 was preferred most frequently, TF2-12B ranked second and consistently above the untuned Gemma-3-12B-it baseline, which received substantially lower scores in fluency and lexical naturalness.

Representative qualitative observations were also recorded during the assessment. As shown in Table 5, the untuned baseline more frequently produced literal phrasing, lexical inconsistencies, or stylistically flat moral endings, whereas TF2-12B showed clearer gains in idiomaticity, entity preservation, and narrative closure. GPT-o3 outputs were generally perceived as the most polished stylistically.

**Table 5:** Illustrative examples of qualitative observations from the human evaluation substudy.

Evaluation aspect	System	Representative observation
Idiomatic phrasing	Baseline	Literal or awkward Romanian rendering of expressions such as “Sharing is caring.”
	TF2-12B	More natural and idiomatic phrasing closer to native usage.
	GPT-o3	Most polished and stylistically refined rendering.
Entity fidelity	Baseline	Occasional species substitutions or corrupted lexical references.
	TF2-12B	Better preservation of animal identities and more natural wording.
Narrative closure	Baseline	Semantically correct but stylistically flat moral endings.
	TF2-12B	Stronger narrative closure and more natural moral tone.

Because only one evaluator was available, this substudy should be interpreted as exploratory evidence rather than definitive human validation. Its primary purpose is to assess whether automated rankings are directionally consistent with informed human judgment. Future work should extend this protocol to multiple independent annotators and report formal inter-rater reliability statistics.

### 4.5 Model comparison and discussion

All TF2 models outperform their untuned bases. For example, TF2-12B improves from 4.43 (Gemma-3-12B-it) to 4.83 at *T* = 0.0 (and from BLEU 0.0214 to 0.0926), and TF2-4B rises from 3.81 (Gemma-3-4B-it) to 4.74 (BLEU 0.1153). The 1B model improves from 2.02 to 3.75 (+1.73 absolute gain).

Fine-tuning substantially narrows the gap to proprietary systems under the rubric-based evaluation, particularly on Style and Cultural/Pragmatic dimensions. Lower BLEU values for some settings are consistent with the stylistic variation and paraphrasing typical of fables, which n-gram overlap metrics may penalize. Across models, 8-bit quantization yields similar rubric scores to FP16, supporting efficient deployment on commodity hardware.

Overall, TF2 shows that parameter-efficient domain adaptation, combined with careful decoding, enables compact open models to achieve strong performance on English–Romanian literary translation under the evaluation setting used in this paper. Representative excerpts are provided in Supplementary Appendix B.

### 4.6 Cost analysis

A central motivation for TF2 is to study English → Romanian literary translation under practical cost constraints. Commercial LLM APIs typically charge per token; at TF2 scale this becomes a major constraint. In our setting, each fable contains roughly 300–600 input tokens and the translation contains 300–600 output tokens, so end-to-end generation for the full TinyFabulist corpus (3M fables) spans *1.8–3.6B tokens* overall.

Table 6 reports estimated costs for translating the full corpus using representative proprietary models, compared to our fine-tuned open TF2 model. We report the mid-case (450 in / 450 out ⇒ 2.7B tokens) and show the low/high ranges in parentheses (300/300 and 600/600). For reasoning models (o3, o3-mini) we include hidden “thinking” tokens by default, which OpenAI bills as output tokens; we assume a medium-effort setting where thinking tokens ≈ visible output tokens.^9^

**Table 6:** Estimated cost of translating the entire 3M-fable TinyFabulist corpus using proprietary APIs vs. our open fine-tuned TF2 model.

Model	Estimated total cost for 3M fables (USD)
GPT-4.1	$13,500 ($9,000–$18,000)
GPT-4.1-mini	$2,700 ($1,800–$3,600)
GPT-o3 (*med. reasoning*)	$24,300 ($16,200–$32,400)
GPT-o3-mini (*med. reasoning*)	$13,365 ($8,910–$17,820)
DeepL API Pro	$270,000 ($180,000–$360,000)
**TF2 fine-tuned (ours)**	**$350**

Mid-case assumes 450 input and 450 output tokens per fable (2.7B tokens total). Ranges show 300/300 and 600/600 scenarios. For o3/o3-mini we include “thinking” tokens (billed as output) at a 1:1 ratio to visible output by default. Bold value indicates the lowest estimated translation cost.

Under these assumptions, proprietary APIs range from about $1.8k–$3.6k for GPT-4.1-mini and $9k–$18k for GPT-4.1, up to $16.2k–$32.4k for o3 at medium reasoning. In contrast, our open-source TF2 model generated all 3M translations for roughly $350 in compute, corresponding to a reduction of 97–99% depending on the baseline. Throughput and cost are helped by vLLM's KV-cache paging and FlashAttention-2 kernels, which enable larger effective batches with similar latency (Kwon et al., 2023).

#### 4.6.1 Sensitivity for reasoning models

If one dials *low* reasoning (thinking tokens ≈ 0.5 × output), o3 spans $12.6k–$25.2k and o3-mini $6.93k–$13.86k; with *high* reasoning (3 × ), o3 rises to $30.6k–$61.2k and o3-mini to $16.83k–$33.66k. Costs scale linearly with total tokens, so other token budgets can be read off proportionally.

Further details on our cost estimation methodology and hardware setups are provided in Supplementary Appendix A.

## 5 DS-TF2-EN-RO-3M dataset description

The DS-TF2-EN-RO-3M dataset is a large-scale, high-diversity English–Romanian parallel corpus of moral fables, curated for open research in low-resource literary translation and controllable generation. Each record is provided as a JSON object following a transparent, extensible schema, enabling robust benchmarking, reproducibility, and in-depth analysis of translation and generation dynamics, in line with best practices in modern NLP dataset curation (Eldan and Li, 2023; Guan et al., 2022; Nadas et al., 2025a).

### 5.1 Schema and metadata

Each entry in the DS-TF2-EN-RO-3M dataset is stored as a JSON object with two groups of fields:

- Fable Content:

- fable: The original English fable, always ending with an explicit moral.
- translated_fable: The Romanian translation produced by the model.
- source_lang and target_lang: Language codes indicating source and target (here, English and Romanian).
- prompt_hash: SHA-256 hash of the generation prompt, ensuring integrity and deduplication.

- Generation Metadata:

- pipeline_stage: The stage of the pipeline that produced the entry (e.g., translation).
- llm_name: Identifier of the model used for generation (e.g., Hugging Face snapshot path).
- translation_model: Explicit reference to the checkpoint used for translation, ensuring full traceability.
- generation_timestamp: Unix timestamp of the translation event, enabling chronological auditing.

### 5.2 Statistical overview

To support reproducibility and downstream benchmarking, we provide a detailed statistical overview of DS-TF2-EN-RO-3M , highlighting its diversity, consistency, and cost-efficiency.

- Diversity: Prompt construction ensures uniform coverage of main characters, morals, and settings; no single template or theme dominates, unlike many traditional narrative datasets.
- Length: On average, stories are ≈450 tokens per language (≈900 tokens per EN–RO pair) —enforcing length consistency and facilitating model training.
- Quality: All Romanian entries were generated using locally fine-tuned open models (Section 4), achieving high rubric and BLEU scores on held-out test sets (see Tables 2, 3). Spot-checks confirm high fluency, coherence, and cultural adaptation.
- Cost: As all data was generated with open-source models on local hardware, the marginal cost of producing the entire dataset is negligible—removing the principal financial barrier to large-scale corpus creation.

Taken together, these properties position DS-TF2-EN-RO-3M as a high-quality, scalable resource for research in low-resource literary translation and controllable generation, particularly in culturally rich domains.

### 5.3 Format and availability

DS-TF2-EN-RO-3M is released in Hugging Face datasets format (JSONL), with open, MIT licensing. Each sample contains both full narrative text and complete provenance metadata, enabling transparent training, evaluation, and reproducibility.

We provide all prompt templates and thematic lists (characters, morals, settings, etc.), together with data generation and evaluation scripts compatible with Hugging Face datasets/transformers. We also include guidelines for extending the schema or adapting it to new domains and languages.

### 5.4 Applications and community impact

DS-TF2-EN-RO-3M provides a scalable platform for fine-tuning and evaluating MT and story generation models in low-resource or creative domains, studying cross-lingual moral reasoning, narrative style, and domain adaptation, as well as benchmarking the cost, efficiency, and quality of open-source LLMs at scale.

Table 7 summarizes how DS-TF2-EN-RO-3M compares to existing literary and low-resource machine translation resources. Compared to existing bilingual and literary corpora, TF2 distinguishes itself by combining large-scale parallel EN–RO data with moral fables as a consistent narrative genre and rich evaluation support. While general-purpose MT corpora such as Europarl (Koehn, 2005b) or OPUS (Tiedemann, 2012) offer breadth across domains but lack literary grounding, and LoResMT (Silva et al., 2024) provides multi-language coverage for low-resource MT with standard metrics (BLEU, COMET, MQM), they do not target moral or narrative-specific translation. Literary datasets like TRANSCOMP focus on stylistic analysis but without parallel structure, while STORAL emphasizes moral story understanding in monolingual English. TF2 therefore fills a complementary niche: large-scale bilingual moral narratives with automatic and rubric-based evaluation tailored for literary adequacy.

**Table 7:** Comparison of TF2 with existing resources in literary and low-resource machine translation.

Dataset	Genre	Languages	Parallel	Evaluation support
**DS-TF2-EN-RO-3M**	Moral fables	EN–RO	Yes	BLEU (secondary) + 5-dim rubric (automated primary)
**DS-TF1-EN-3M**	Moral fables	EN	No	N/A
TRANSCOMP	Literary (varied)	120EN	No	Stylistic analysis only
LoResMT	General MT	26 low-resource	Yes	BLEU, COMET, MQM
STORAL	Moral stories	EN	No	Human annotation (moral inference, story completion)

Bold values indicate the dataset contributed in this work (DS-TF2-EN-RO-3M).

Availability: Dataset access, code, and documentation are available at https://huggingface.co/datasets/klusai/ds-tf2-en-ro-3m and the TinyFabulist GitHub repository.

## 6 Discussion

This section synthesizes the empirical findings without introducing new experimental results. Our results provide insights into the capabilities and limitations of LLM-based translation for creative content, with a focus on moral fables.

### 6.1 Mapping of results to research questions

To structure our findings, we summarize how each research question is addressed by the results presented in the paper:

- RQ1 (Cost-Constrained Data Generation): Addressed through our empirical analysis of dataset creation (Section 5) and cost benchmarking (Section 4). We show that a large-scale English–Romanian literary translation corpus (DS-TF2-EN-RO-3M ) can be constructed under strict budget constraints using open models, while documenting the trade-offs introduced by synthetic, machine-generated reference translations.
- RQ2 (Open vs. Proprietary Translation Quality): Addressed via comparative benchmarking in Section 4, which contrasts proprietary systems (GPT-4.1, Gemini, DeepL) with open-source models before and after parameter-efficient fine-tuning. The results indicate that fine-tuned open models substantially narrow the gap to proprietary APIs under the evaluation setting used in this paper, while remaining more efficient and deployable.
- RQ3 (Evaluation Rigor and Automation): Addressed through the combined use of an automated LLM-as-a-judge rubric (Section 3.1) and a targeted human evaluation on a representative subset. We find that LLM-based evaluation provides useful and interpretable quality signals for this task, and that system rankings are broadly consistent with expert human judgments in our pilot study, while acknowledging potential sources of bias and the limits of small-scale human annotation.

### 6.2 Quality–cost trade-offs

Across the evaluated baselines, GPT-o3 attains the highest rubric scores and is therefore a natural choice for producing silver-standard reference translations in our pipeline. While GPT-4.1 and GPT-o3 share the same visible token pricing, the effective cost of GPT-o3 can be substantially higher when hidden reasoning tokens are billed as output tokens (Section 4.6). These results underscore the importance of benchmarking translation systems in the target domain and under a transparent cost model, rather than selecting systems based solely on model size, recency, or nominal token prices.

### 6.3 Open-source model potential

Off-the-shelf open-weight models, especially at smaller scales (e.g., Gemma-3-1B-it), show limited performance on English–Romanian literary translation without adaptation. Parameter-efficient fine-tuning with LoRA on the DS-TF2-EN-RO-15K corpus yields consistent improvements under both BLEU (relative to the GPT-o3 reference) and rubric-based evaluation, most notably for the 4B and 12B models. At the same time, under the evaluation setting used here, proprietary systems remain stronger overall. The practical value of the adapted open models lies in their deployability: they can be hosted locally, quantized with limited loss under the rubric, and used in cost-constrained environments where API-based translation is impractical.

### 6.4 Error patterns in literary translation

Qualitative inspection and rubric rationales indicate that even top-performing systems occasionally produce overly literal renderings, unusual lexical choices, or minor syntactic artifacts. Lower-capacity or untuned models exhibit more frequent omissions, awkward phrasing, and agreement errors (e.g., gender or tense mismatches in Romanian). These observations are consistent with the general challenge of evaluating and optimizing MT for creative domains, where fidelity, style, and discourse structure interact in non-trivial ways. They further motivate domain-adaptive fine-tuning and evaluation protocols that consider more than surface-level lexical similarity.

### 6.5 LLM-as-a-judge evaluation

Our evaluation pipeline uses GPT-o3-mini as an automated judge to provide multi-dimensional scoring of translation outputs across accuracy, fluency, coherence, style, and cultural adaptation. In addition, we include a cross-family judge check (Section 4) and a targeted human evaluation substudy to anchor the interpretation of automated scores. Prior work suggests that rubric-based LLM evaluation can provide useful comparative signals in text generation tasks (Liu et al., 2023). In our setting, automated rankings broadly align with the human pilot study, but reliance on any single judge (or family of judges) may still encode systematic preferences. Future work should expand the human evaluation set, include multiple independent judges, and explore alternative quality-estimation signals designed specifically for translation.

### 6.6 Domain and genre considerations

Fables offer a useful setting for studying literary translation: they are short, narrative, and require preservation of both story content and moral framing, while remaining easier to scale than longer-form literature. At the same time, their relatively structured form may make them more tractable than genres with denser metaphor, dialogue, or culturally embedded references. Consequently, the results reported here should be interpreted as evidence for the feasibility of cost-efficient pipelines in this genre and language pair, rather than as claims of equivalence with professional human translation quality across literary domains.

### 6.7 Summary

Overall, TF2 illustrates that a cost-aware pipeline combining synthetic data generation, parameter-efficient fine-tuning, and open benchmarking can yield strong English–Romanian literary MT systems under practical constraints. While open models do not match the strongest proprietary systems under our evaluation setting, fine-tuning and careful decoding substantially improve performance, and quantized checkpoints remain competitive under rubric-based assessment. We release the datasets, models, and codebase to support follow-up work on broader genres, stronger human evaluation protocols, and more robust translation-specific evaluation methods.

## 7 Threats to validity and limitations

No evaluation is without limitations. We outline several threats to the validity of our results and claims:

Bias in LLM-Based Evaluation: Relying on a single LLM (GPT-o3-mini) as the judge for translation quality may introduce bias. If this evaluator has particular preferences or weaknesses (e.g., favoring literal renderings or being too lenient on certain errors), then scores could systematically misrepresent quality. This concern is amplified because our silver-standard references are themselves generated by a GPT-o3 variant, which raises the possibility of family favoritism.

To mitigate this, we performed a targeted *cross-family judge bias check* by re-scoring an identical 100-item subset with Grok-3-mini, an unrelated model family, under the same rubric and randomized setup (Table 4). The ranking of systems remained stable across judges, and the TF2–o3 performance gaps were actually smaller under Grok-3-mini, giving us increased confidence that our conclusions are not an artifact of evaluator bias. That said, this single cross-check cannot fully rule out all sources of bias. Our results suggest consistency across families, but we caution that automatic LLM-based scoring remains a proxy. Broader validation with human evaluators or a diverse panel of LLM judges would provide stronger guarantees.

Ground Truth Translations Are Synthetic: Our reference “ground truth” Romanian texts come from GPT-o3, not from human translators. This has multiple implications. First, these references might contain subtle errors or unnatural phrasing. If a system happened to produce a more natural translation that deviates from the reference, metrics like BLEU would unfairly penalize it. Similarly, the LLM judge might sometimes incorrectly label a perfectly good translation as wrong because it differs from the GPT-o3 phrasing (this is a known issue with reference-based evaluations). We attempted to reduce this effect by focusing the LLM evaluator on meaning and not exact wording, but it's impossible to eliminate entirely. In short, our benchmark measures similarity to a very strong machine translation, which is not exactly the same as similarity to a human translation or fidelity to the author's intent. The use of a single reference translation is a general limitation in MT evaluation—multiple reference translations (if available) or more nuanced metrics could address this.

Generality to Other Domains: Our findings are specific to the domain of fables. Fables have fairly straightforward language and repetitive structure. This might inflate the perceived ability of models; for example, models can often translate formulaic sentences or moral lessons consistently once they catch the pattern. In more complex literary texts (say, a novel with sarcasm, historical dialogue, or poetry), the performance of both our data generation approach and the models themselves might be much weaker. Thus, one should be careful in extending the conclusions to “literary translation” in general. We believe the cost-effective methodology would still apply, but quality outcomes might vary widely by genre.

Model Obsolescence and Heterogeneous Setups: By the time of publication, there may be newer versions of models (GPT-4.2, Gemini 3, etc.) or completely new contenders. The specific numeric results we report (scores, BLEU) are tied to the snapshot of models as of early 2025. Additionally, the models were used under different conditions: GPT-4.1 and GPT-o3 via API, Gemini via a research preview, DeepL via its API, open models on our hardware. Each has constraints (context window limits, etc.) that we normalized as best as possible (we ensured the full fable input fit in every model's context). Small differences like using temperature 0 for some models vs. their default might also affect outputs. We chose settings to optimize quality for each model individually, which we believe is fair, but this means some models might have had advantages (e.g., if one model performs better with slight randomness, we could in theory sample multiple outputs and choose the best—we did not do that except regenerating obvious failures). The upshot is that our benchmark results are illustrative rather than absolute. We encourage others to rerun the evaluation with future models or different decoding strategies; our code and data allow for that.

Scale of Human Evaluation: We evaluated 100 test samples with the LLM judge for each model. This is a limited subset (2% of the test set). It is possible that had we evaluated all 1,500 test stories with the LLM (at considerable cost and time), the averages might shift slightly. We assumed 100 random samples would be representative enough for a comparative judgment, and it provided us with quick qualitative feedback. The BLEU scores, on the other hand, are on the full test set. There could be minor inconsistencies—for example, if the 100 stories were on average simpler or harder than the rest, that could skew the scored results. We tried to mitigate this by truly random selection. In future or in a finalized version of the benchmark, we could increase the sample size of judged translations, or use stratified sampling to ensure covering various story types.

Reproducibility of Cost Claims: Our cost analysis is based on API pricing and hardware efficiency at the time of our experiments, but these figures are subject to change as providers update rates or infrastructure. The estimated costs for GPT-o3 and GPT-4.1 are derived from OpenAI's published token pricing, and real-world expenses may vary depending on prompt length, output size, and billing practices (such as counting reasoning tokens). Similarly, Google Gemini's pricing may fluctuate and may differ by region or access tier. Fine-tuning the Gemma-3-1B model incurs an additional one-time training cost—typically a few hours on a modern GPU—which we did not include in the per-translation inference cost but is relevant to the overall project budget. We encourage other researchers to recalculate all costs using current token rates, hardware prices, and their own data characteristics when planning similar experiments.

In summary, while our benchmark and findings provide a useful data point and methodology for low-resource translation, they should be interpreted with an understanding of these limitations. We believe the overall trends—such as the viability of synthetic data and the strong performance of fine-tuned small models—are robust, but exact numbers and minor rankings could vary under different conditions. We encourage the community to treat TF2 as a starting point, to be refined and validated with further human studies and extended to other contexts.

## 8 Conclusion

We have presented TF2 , a comprehensive benchmark and dataset for English-to-Romanian literary translation, with a focus on cost-constrained use of AI models. In this paper, we detailed how a large parallel corpus of fables was automatically constructed using a modest budget and leveraged to evaluate a range of translation systems. Our results show that modern LLMs can produce translations of creative text with remarkable quality—approaching strong expert-perceived quality under the present evaluation framework in many cases—even for a language like Romanian that traditionally lacks extensive MT resources. Moreover, by carefully balancing cost and performance, we demonstrated that it is possible to achieve these results without exclusive reliance on expensive proprietary models: open-source models, when fine-tuned on the right data, can close much of the quality gap while essentially eliminating per-translation costs. A small pilot human evaluation broadly supported the automated ranking trends, while also highlighting the need for larger multi-annotator studies.

The TF2 project makes several contributions to the community: the open release of a 15k fable translation dataset, an evaluation toolkit combining multidimensional rubric assessment with BLEU reported as a secondary reference-based consistency metric, and reproducible pipelines for cost analysis and model fine-tuning. We believe these resources will be useful not only for benchmarking translation models but also for broader research into low-resource NLP, narrative understanding, and the interplay between synthetic data and model training. For instance, one immediate application of our dataset is to train a bespoke Romanian fable generation model (analogous to TinyFabulist TF-4 in our series roadmap) or to study how exposure to moral stories in two languages might help a model's reasoning or cross-lingual abilities. Future Romanian generation models trained on synthetic translated corpora should also be monitored for propagation of translation artifacts, motivating hybrid human-curated extensions.

In terms of future work, several directions emerge. First, extending the methodology to other language pairs and genres: could we create a TinyFabulist for, say, Swahili or Vietnamese fables? How about translating folk tales or poems? Each would pose new challenges for LLMs and might require refining the prompt strategies or using different reference models. Second, incorporating human feedback: an interesting follow-up would be to have bilingual human evaluators rank a sample of translations from each model, to verify our LLM-judge findings. A small pilot human evaluation in the present study broadly supported the automated ranking trends, while also highlighting the need for larger multi-annotator studies. Such future work could also facilitate fine-tuning the evaluation LLM (via reinforcement learning from human feedback, RLHF) to better align with human judgments. Third, exploring model fine-tuning more deeply: our work fine-tuned a 1B model out-of-the-box. It would be worthwhile to experiment with larger open models (e.g., 7B or 13B Llama variants) fine-tuned on the TF2 data, which might substantially improve quality and approach the performance range of larger proprietary systems under our evaluation setting.

Finally, we plan to integrate the lessons from TF2 into training a fully open Romanian fable generator (TinyFabulist TF-3). By having a translated corpus, we can also explore cross-lingual training—perhaps teaching a model to generate fables directly in Romanian by leveraging English data as well. Such a model could become one of the first openly reproducible systems designed to natively produce Romanian moral stories at scale. This ties back to our broader goal: enabling AI systems that can serve local cultural needs, such as Romanian educational content, without being gated by access or cost. TF2 is a step in that direction, illustrating that with careful engineering, the benefits of large language models can be brought to low-resource scenarios in a practical and open manner. Notably, the full 3M-fable corpus was produced for approximately $350 in compute cost, illustrating the practical accessibility of such pipelines.

## Data Availability

The datasets presented in this study can be found in online repositories. The names of the repository/repositories and accession number(s) can be found below: https://huggingface.co/datasets/klusai/ds-tf2-en-ro-3m https://huggingface.co/datasets/klusai/ds-tf2-en-ro-15k.

## Footnotes

1 WMT stands for the Conference on Machine Translation (formerly “Workshop on Machine Translation”). The WMT organizes annual shared tasks for benchmarking machine translation systems, with its “news translation” task being the most widely used standard evaluation for translation of contemporary news articles. See: https://www.statmt.org/wmt23/.

2 https://huggingface.co/datasets/klusai/tf2-en-ro-15k, https://huggingface.co/datasets/klusai/tf2-en-ro-3m

3 https://huggingface.co/klusai/tf2-1b, https://huggingface.co/klusai/tf2-4b, https://huggingface.co/klusai/tf2-12b

4
https://huggingface.co/datasets/klusai/ds-tf1-en-3m

5
https://huggingface.co/datasets/klusai/tf2-en-ro-15k

6
https://github.com/ggml-org/llama.cpp

7
https://huggingface.co/datasets/klusai/tf2-en-ro-3m

8 Evaluation setup as in Section 3.1 (100 fables per model, five-dimension rubric, JSON outputs).

9 OpenAI bills hidden reasoning (“thinking”) tokens as *output* tokens; the amount depends on the requested reasoning_effort.
