# Supplementary material for: Building large-scale English–Romanian literary translation resources with open models
Source: Front Artif Intell. 2026 Jun 17;9:1807431. doi: 10.3389/frai.2026.1807431 (PMC13318819; doi:10.3389/frai.2026.1807431)
Supplement: Supplementary file 1 [file Supplementary_file_1.docx]

**Supplementary Material**

*Building Large-Scale English–Romanian Literary Translation Resources with Open Models*

Mihai Nadaș, Laura Dioșan, Andreea Tomescu, and Andrei Pișcoran

Frontiers in Artificial Intelligence · Article ID 1807431 · DOI 10.3389/frai.2026.1807431

# **Supplementary Appendix A. Cost Calculation and Hardware Configurations**

## **A.1 Cost Estimation Methodology**

Supplementary Table A1 details the assumptions used to compute cost estimates in Section 4.6. We model cost as a function of input tokens (T_in), output tokens (T_out), and API pricing per million tokens. For proprietary reasoning models (e.g., GPT-o3), we assume hidden reasoning tokens are billed as output, with a “medium reasoning” setting (reasoning tokens ≈ visible output).

*Total Cost = (T_in / 10⁶) × P_in + (T_out + T_reason) / 10⁶ × P_out*

**Supplementary Table A1. Per-million-token pricing assumptions used in cost calculations.**

| **Model** | **P_in ($/M)** | **P_out ($/M)** | **Notes** |
| --- | --- | --- | --- |
| GPT-4.1 | 2.00 | 8.00 | Standard API pricing (Aug 2025) |
| GPT-4.1-mini | 0.40 | 1.60 | Lower capacity, same billing rules |
| GPT-o3 | 2.00 | 8.00 | Reasoning tokens billed as output |
| GPT-o3-mini | 1.10 | 4.40 | Same reasoning token policy |
| DeepL API Pro | — | — | Flat monthly + per-character rate, converted to tokens |
| TF2 models (ours) | — | — | Rented GPUs on sfcompute (≈ $350 for 3M fables; see A.3 for the exact figure) |

## **A.2 Hardware Configurations for TF2 Models**

Supplementary Table A2 lists the compute environments used for training and inference. We relied exclusively on commodity GPUs (cloud or local) with support for FP16/bfloat16 mixed precision and 8-bit quantization (W8A8).

Two distinct compute regimes were used. **Stage 1 baselines (the proprietary and open instruction-tuned systems compared in Section 4.1)** were queried via the OpenRouter API, which provided unified access to both open-weight and proprietary models for the 100-item rubric evaluation. **Mass translation of the full 3M-fable corpus (Stage 4) was carried out on self-hosted infrastructure**: rented 8×H100 nodes on sfcompute serving vLLM endpoints with our fine-tuned TF2 checkpoints. The $350 cost figure reported in Section 4.6 and Table 6 refers exclusively to this self-hosted Stage 4 run; the OpenRouter calls used for Stage 1 baselines are separate and not included in that figure.

**Supplementary Table A2. Hardware setups for training and inference.**

| **Stage** | **Hardware** | **Runtime** | **Notes** |
| --- | --- | --- | --- |
| Stage 1 baseline evaluation | OpenRouter API | — | 100-item rubric over 13 baseline systems (Section 4.1); not included in the $350 cost figure |
| Fine-tuning TF2-1B | 1 × L40S | 1h | LoRA adapters, FP16 |
| Fine-tuning TF2-4B | 1 × L40S | 2–3h | Gradient accumulation, FP16 |
| Fine-tuning TF2-12B | 1 × H100 | 2h | FP16, early stopping |
| Inference — mass translation of 3M fables (Stage 4) | 8 × H100 | ~32h | sfcompute clusters, vLLM endpoints; the $350 cost figure in Section 4.6 corresponds to this run |
| Quantization | CPU + GPU mix | <1h/model | W8A8 with llmcompressor |

## **A.3 Energy and Cost of Local Compute**

To approximate the cost of our Stage 4 mass translation run, we base calculations on the provider we used (sfcompute):

- GPU rental: $1.35 per GPU-hour; our 8-GPU node is billed at $10.80 per cluster-hour.
- Electricity: Not applicable (cloud rental includes energy). For on-prem scenarios only, a rough estimate is 300–450 W/GPU at $0.14/kWh (≈ $0.04–$0.06 per GPU-hour).

*C_rental [$] = 10.8 × H_cluster*

where H_cluster is the logged wall-clock cluster time (hours). Our Stage 4 mass translation run used **32 cluster-hours, for a billed cost of $345.60**, which is reported throughout the paper as “≈ $350” (rounded). For reference, 24 and 40 cluster-hours would correspond to $259.20 and $432.00, respectively. Storage and network costs were negligible in our runs.

## **A.4 Software Environment**

Experiments were conducted in the following software stack:

- **OS:** Manjaro + Linux 6.12
- **Frameworks:** PyTorch 2.7.1, Hugging Face Transformers 4.54.0, PEFT 0.11
- **Inference:** vLLM 0.7.3, FlashAttention
- **Quantization:** llmcompressor (Aug 2024 release)
- **Deployment artifacts:** GGUF and Safetensors formats

# **Supplementary Appendix B. Qualitative Translation Examples (Five Cases)**

We present in Supplementary Table B1 five representative problem cases. For each, we show excerpts from the Original (EN), the Gemma-3-12B (untuned) output, and the TF2-12B (fine-tuned) output. We highlight in bold the key segments where fine-tuning clearly improves lexical choice, species fidelity, and idiomatic Romanian.

**Supplementary Table B1. Excerpts from five difficult fables. TF2-12B preserves correct species and natural idioms, while Gemma-3-12B often introduces species corruption, typos, or ungrammatical forms.**

| **Original (EN)** | **Gemma-3-12B (untuned)** | **TF2-12B (fine-tuned)** |
| --- | --- | --- |
| *“A* ***Greedy Skunk*** *… ‘I’m special because I’m the* ***best treasure hunter****!’”* | *„Un* ***Fumeg de lăcomie*** *… ‘Sunt special …* ***căutător de comori****!’”* | *„****Sconcsul Lacom*** *… ‘Sunt special …* ***vânător de comori****!’”* |
| *“… a kind and gentle* ***cheetah*** *…* ***plant a strong tree*** *…* ***lasting loyalty****.”* | *„…trăia o* ***ceată blândă*** *… loialitate de durată.”* | *„…trăia o* ***ghepardă blândă*** *… simbol al noii prietenii … loialitate de durată.”* |
| *“****The Hippopotamus’ Hidden Treasure*** *… ‘****Sharing is caring****.’ …* ***light the way****.”* | *„****Iepurașul și Comoara Ascunsă*** *… ‘Împărtășirea …’ … lumina drumul.”* | *„****Comoara ascunsă a hipopotamului*** *… ‘A împărți înseamnă a-ți păsa.’ … lumina calea.”* |
| *“… a clever* ***skunk*** *… a* ***butterfly*** *… grumpy old* ***owl*** *…* ***reflections*** *…”* | *„… un pui de* ***prepeliță*** *… o* ***libelulă*** *… un* ***bufnițel*** *…”* | *„… un* ***sconcs*** *… un* ***fluture*** *… o* ***bufniță*** *… propriile reflexii …”* |
| *“… a greedy* ***puma*** *… ‘power of* ***teamwork****.’”* | *„… un* ***pumnă malign*** *… puterea muncii în echipă.”* | *„… un* ***puma*** *lacom … puterea muncii în echipă.”* |

*Note: Bold segments mark the key differences where fine-tuning improves lexical choice, species fidelity, and idiomatic Romanian.*
